# Additive interactions between obesity and insulin resistance on hypertension in a Chinese rural population

**DOI:** 10.1186/s12889-023-17454-1

**Published:** 2023-12-15

**Authors:** Xiaoxia Li, Xiaoyu Chang, Yuanyuan Dang, Yixuan Xue, Qingan Wang, Wanlu Liu, Ting Yin, Yi Zhao, Yuhong Zhang

**Affiliations:** 1 Department of Epidemiology and Health Statistics, School of Public Health, Ningxia Medical University, Yinchuan, 750004 China; 2 Key Laboratory of Environmental Factors and Chronic Disease Control, School of Public Health of Ningxia Medical University, Yinchuan, 750004 China; 3 Editorial Board of Journal of Ningxia Medical University, Yinchuan, 750004 China; 4 Department of Nutrition and Food Hygiene, School of Public Health of Ningxia Medical University, Yinchuan, 750004 China

**Keywords:** Obesity, Body mass index, Waist circumference, Body fat percentage, Insulin resistance, Hypertension

## Abstract

**Background:**

Adiposity and insulin resistance (IR) are closely associated with hypertension; however, the role of interactions between obesity phenotypes and IR in hypertension is unclear. This study aimed to evaluate the interactions of body mass index (BMI), waist circumference (WC), and body fat percentage (BF%) with IR on hypertension risk.

**Methods:**

We analyzed data from 4888 participants (mean age 57 years, 41.2% men) in the China Northwest Natural Population Cohort, Ningxia Project. BMI, WC, and BF% were determined using bioelectrical impedance analysis devices. IR was estimated using a homeostasis model assessment index (HOMA-IR). Multivariable-adjusted logistic regression was used to evaluate the association between HOMA-IR and hypertension risk. We calculated the relative excess risk and attributable proportion with their 95% confidence intervals (CIs) to assess whether adiposity phenotypes modified the effect of HOMA-IR on hypertension risk.

**Results:**

The crude prevalence of hypertension was 52.2%. The multivariable-adjusted odds ratio of HOMA-IR was 1.80 (95% CI: 1.23–2.65) for the risk of hypertension in the highest versus the lowest quartiles, but this association became marginal in models further adjusting for BMI, WC, and BF% (P for trend = 0.056). Relative excess risk and attributable proportion for interaction between high HOMA-IR and high BF% were 0.32 (0.04–0.59) and 0.33 (0.06–0.60), respectively. Additionally, high truncal and leg BF% and high HOMA-IR accounted for the hypertension risk in women, but not in men. We did not observe any significant interactions between BMI or WC and HOMA-IR on hypertension.

**Conclusion:**

BF% modified the association between IR and increased risk of hypertension in women with high truncal and leg BF%, but not in men.

**Supplementary Information:**

The online version contains supplementary material available at 10.1186/s12889-023-17454-1.

## Background

The global prevalence of hypertension is rising. In 2019, approximately 12.78 million people worldwide had hypertension [1], leading to a large global burden of cardiovascular disease and premature death [2]. Previous studies have demonstrated that insulin resistance (IR) [3], obesity [4], and hypertension are closely interrelated.

Different measures of obesity have been defined, including the well-recognized body mass index (BMI) reflecting total body mass[BMI~(weight/height**2)], waist circumference (WC) reflecting abdominal obesity, and body fat percentage (BF%). Considerable evidence from longitudinal studies confirms that WC and BMI are significantly associated with an increased risk of hypertension in diverse populations [4, 5]. Moreover, obesity plays a role in the pathogenesis of IR [6, 7]. As summarized in a meta-analysis, obesity indicators such as WC and BMI were most commonly used for variable adjustments when analyzing the relationship between IR and hypertension [3]. Some researchers found that obesity may have a mediating effect on the association between IR and hypertension [8, 9], but others reported that obesity does not modify this relationship [10]. Therefore, the role of obesity in the relationship between IR and hypertension based on the results of existing studies remains controversial. Recent studies have suggested that BF% is significantly associated with risk of hypertension [11, 12]. It is worth noting that a Korean cohort study revealed BF% as a predictor of hypertension, even in nonobese individuals who were defined based on BMI and WC criteria [13]. However, no study has evaluated the role of BF% on the association between IR and hypertension. IR and obesity are risk factors for hypertension, that often coexist. Only a few studies have reported an interaction between obesity and IR in hypertension. A review reported that obesity, particularly if defined by WC, appears to have an important influence on the IR–hypertension relationship [14], and a study in postmenopausal women revealed a significant interaction between WC and log homeostasis model assessment-IR (HOMA-IR) on systolic blood pressure (SBP) [15]. Results from a prospective cohort study, using IR to define unhealthy metabolic statuses, found that overweight and obese participants in IR groups showed a significant and independent risk of hypertension [16]. Zhang et al. [17] have shown that adiposity in the development of hypertension is modified by IR. Therefore, obesity and IR are interconnected in various ways. The role of the interactions between different obesity indicators and IR on hypertension risk remains unclear.

Although the coexistence of IR and obesity as major risk factors for hypertension has been widely documented, Chinese rural populations may show different associations. Herein, we aimed to assess the association between IR and hypertension in a Chinese rural population and further explored the interactions between different obesity indicators (WC, BMI, and BF%) and IR on hypertension risk.

## Materials and methods

### Study population

This study was derived from the China Northwest Natural Population Cohort, Ningxia Project (CNC-NX), an ongoing population-based prospective cohort study. The CNC-NX protocol has been previously reported in detail [18]. Briefly, 15,802 participants (age range 35–74 years) from 45 villages in Wuzhong and Shizuishan City in the Ningxia Hui Autonomous Region of China were enrolled at baseline between March 2018 and May 2019. Demographic characteristics and anthropometric and biochemical measurements were obtained from all participants. Of these 15,802 participants, approximately 30% (5300 participants) were randomly selected comprising a representative subcohort. Among these 5300 individuals, we excluded 73 with missing data on fasting insulin levels, 117 with missing lipid parameters, 75 with missing blood pressure data, and 147 with other missing variables. Therefore, 4888 participants were included in our analysis.

### General information and anthropometric measurements

Information on general characteristics (e.g., age, sex, educational status, cigarette smoking, alcohol intake, disease history, medication history) was extracted by trained research assistants from questionnaires. The body height was measured without shoes. BMI, WC, and BF% were estimated using bioelectrical impedance analysis (BIA) devices (InBody 370 system, Biospace, Korea) according to standard operating guidelines. The participants removed outer garments and stood barefoot on the BIA device, which passed small electrical currents through the body to estimate body composition. SBP and diastolic blood pressure (DBP) were measured using an OMRON automatic monitor (OMRON-7124, Omron Corporation, Japan) after the participants had rested for at least 5 min. Two consecutive readings were obtained and the average of the two readings was calculated as the blood pressure value for analysis.

### Biochemical measurements

Blood samples were collected from the participants between 6:00 and 8:00 a.m. after 8–12 h of fasting. Biochemical indicators were measured using a biochemical autoanalyzer (Mindray BS-430, Shenzhen, China). Fasting insulin levels were measured using a chemiluminescence immunoassay analyzer (Mindray CL-2000i, Shenzhen, China). IR was estimated using the HOMA-IR and calculated using the following formula: HOMA-IR = fasting glucose (mmol/L) × fasting insulin (mIU/mL)/22.5 [19].

### Definitions

Hypertension was defined as SBP ≥ 140 mmHg, DBP ≥ 90 mmHg, self-reported hypertension, or current use of blood pressure-lowering medication. Obesity was defined as: (1) WC ≥ 90 cm for men and ≥ 85 cm for women, (2) BMI ≥ 30 (kg/m^2^), and (3) BF% ≥25% in men and ≥ 35% in women [20]. IR was defined in the present study as an HOMA-IR value above the 75th percentile (> 2.59).

### Statistical analysis

Participant characteristics were described according to the presence or absence of hypertension. Except for insulin levels and HOMA-IR, which are presented as the median, other continuous variables are presented as the mean ± standard deviation. Nonparametric tests or t-tests were used to compare differences between the non-hypertension and hypertension groups, where appropriate. Categorical variables such as sex, education status, cigarette smoking, and alcohol intake are presented as frequencies (%) and were compared using the chi-square test. A binary logistic regression model adjusted for related potential confounders was used to examine the association between HOMA-IR and hypertension, especially in Model 4 with added WC, BMI, and BF%, to test whether different obesity indicators affect the relationship between HOMA-IR and hypertension. Test for trends based on variables containing median values for each quartile. In addition, we assessed the additive interaction based on Hosmer et al. [21] to calculate the relative excess risk (RERI) and attributable proportion (AP) due to interaction if the 95% confidence interval (CI) did not include 0, suggesting that significant interactions exist. These parameters have been proposed as interaction measures in epidemiologic studies [22]. All data were analyzed using SPSS (version 23.0; IBM Corp, Armonk, NY, USA) and R software (The R Foundation, Vienna, Austria), and *P*-values < 0.05 were considered statistically significant.

## Results

### Baseline characteristics of the study participants

We included 4888 participants with a mean age of 57.38 years. The characteristics of the hypertension and non-hypertension groups are shown in Table 1. Compared to participants with hypertension, those without hypertension tended to be younger (mean age: 60.03 vs. 54.49 years). Participants with hypertension had higher HOMA-IR, WC, BMI, WHR, BF%, SBP, and DBP, as well as higher TG, TC, fasting blood glucose (FBG), and fasting insulin levels, than those without hypertension (all *P* < 0.001). There were no significant differences in LDL-cholesterol levels, sex, current smoking status, and current drinking status between the two groups.

**Table 1:** Characteristics of the study participants

Characteristics	Total (n = 4888)	Hypertension (n = 2552)	Non-hypertension (n = 2336)	*P-*value
Age (years)	57.38 ± 9.93	60.03 ± 9.07	54.49 ± 10.03	< 0.001
Male sex, n (%)	2013 (41.2)	1083 (22.2)	930 (39.8)	0.062
Current smoking, n (%)	525 (10.7)	263 (10.3)	262 (11.2)	0.305
Current drinking, n (%)	265 (5.4)	144 (5.6)	121 (5.2)	0.475
Education status, n (%)				
Illiteracy/primary school	3385 (69.3)	1881 (73.7)	1504 (64.4)	< 0.001
Middle or higher school	1403 (31.3)	671 (26.3)	832 (35.6)	
FBG (mmol/L)	6.02 ± 2.61	6.53 ± 2.91	6.13 ± 3.05	< 0.001
Fasting insulin (µIU/mL)	6.50 (4.64–9.38)	6.98 (4.99–9.97)	6.00 (4.29–8.53)	< 0.001
HOMA-IR	1.66 (2.59–1.15)	1.89 (1.26–2.88)	1.49 (1.03–2.27)	< 0.001
WC (cm)	87.37 ± 9.91	89.59 ± 10.16	85.28 ± 9.13	< 0.001
BMI (kg/m^2^)	25.09 ± 3.50	25.80 ± 3.54	24.31 ± 3.28	< 0.001
WHR	0.92 ± 0.06	0.94 ± 0.06	0.91 ± 0.06	< 0.001
BF%	31.97 ± 7.69	33.37 ± 7.44	30.44 ± 7.67	< 0.001
SBP (mmHg)	138 ± 19	148 ± 17	122 ± 11	< 0.001
DBP (mmHg)	83 ± 13	91 ± 12	75 ± 8	< 0.001
TG (mmol/L)	1.78 ± 1.24	1.89 ± 1.31	1.67 ± 1.14	< 0.001
TC (mmol/L)	4.90 ± 1.30	4.98 ± 1.52	4.81 ± 1.00	< 0.001
HDL-cholesterol (mmol/L)	1.35 ± 0.41	1.34 ± 0.35	1.37 ± 0.46	0.004
LDL-cholesterol (mmol/L)	2.86 ± 0.87	2.87 ± 0.84	2.86 ± 0.89	0.626

The values are presented as mean ± standard deviation, median (interquartile range), or number (%)

*BF%* body fat percentage; *BMI* body mass index; *DBP* diastolic blood pressure; *FBG* fasting blood glucose; *HDL* high-density lipoprotein; *HOMA-IR* homeostasis model assessment-insulin resistance; *LDL* low-density lipoprotein; *SBP* systolic blood pressure; *TC* total cholesterol; *TG* triglyceride; *WC* waist circumference; *WHR* waist-to-hip ratio

### Multivariable-adjusted logistic regression analysis of HOMA-IR and hypertension

Table 2 shows the associations between HOMA-IR and hypertension using multivariable logistic regression analyses. HOMA-IR was divided into quartiles (Q1–Q4) with Q1 as the reference. After adjustment for age, sex, educational status, cigarette smoking, drinking, history of hypertension and diabetes mellitus, FBG, SBP, total cholesterol, and triglyceride in Model 3, the odds ratios (ORs) for hypertension compared to HOMA-IR Q1 were 1.11 (95% CI: 0.80–1.56) for Q2, 1.48 (95% CI: 1.04–2.09) for Q3, and 1.80 (95% CI: 1.23–2.65) for Q4 (P for trend = 0.001). However, the association between HOMA-IR and hypertension risk was not significant after further adjustment for WC, BMI, and BF% (Model 4; P for trend = 0.056). Similar associations were observed when HOMA-IR was included as a continuous variable.

**Table 2:** Multivariable-adjusted logistic regression analysis of HOMA-IR in relation to hypertension

	Model 1 OR (95% CI)	Model 2 OR (95% CI)	Model 3 OR (95% CI)	Model 4 OR (95% CI)
HOMA-IR	1.12 (1.08–1.16)	1.08 (1.04–1.12)	1.05 (1.01–1.10)	1.04 (0.99–1.06)
HOMA-IR (quartiles)				
Q1 (< 1.15)	1.00 (ref)	1.00 (ref)	1.00 (ref)	1.00 (ref)
Q2 (1.15–1.66)	1.32 (1.12–1.54)	1.65 (1.35–2.00)	1.11 (0.80–1.56)	1.02 (0.72–1.44)
Q3 (1.67–2.59)	1.88 (1.61–2.21)	2.41 (1.98–2.94)	1.48 (1.04–2.09)	1.28 (0.88–1.85)
Q4 (> 2.59)	2.36 (2.01–2.77)	2.61 (2.13–3.20)	1.80 (1.23–2.65)	1.46 (0.95–2.25)
P for trend	< 0.001	< 0.001	0.001	0.056

Data are presented as ORs and 95% CIs.

Model 1: crude

Model 2: adjusted for age, sex, educational status, cigarette smoking, drinking, history of hypertension and diabetes mellitus

Model 3: adjusted for all the factors in Model 2 and FBG, SBP, TC, and TG.

Model 4: adjusted for all the factors in Model 3 and WC, BMI, and BF%.

Test for trend based on the variable containing the median value of each quartile

*BF%* body fat percentage; *BMI* body mass index; *CI* confidence interval; *FBG* fasting blood glucose; *HOMA-IR* homeostasis model assessment-insulin resistance; *OR* odds ratio; *SBP* systolic blood pressure; *TC* total cholesterol; *TG* triglyceride; *WC* waist circumference

### Association between HOMA-IR and risk of hypertension according to WC, BMI, and BF%

We stratified participants by WC, BMI, and BF% to further explore whether different obesity indicators play a mediating role in the relationship between IR and hypertension (Table 3). After adjusting for potential confounders, the relationship between HOMA-IR and hypertension risk was stronger (OR = 1.91, 95% CI: 1.10–3.31, *P* = 0.022) among participants with higher BF% than among those with normal BF%. However, after stratification according to BMI or WC, the relationship between HOMA-IR and hypertension was not significant after adjusting for potential confounders.

**Table 3:** Association of HOMA-IR and risk of hypertension according to WC, BMI, and BF%

	No. of participants	OR (95% CI)	*P*-value	No. of participants	OR (95% CI)	*P*-value	P-interact
HOMA-IR (quartiles)	WC (cm): men ≥ 90, women ≥ 85			WC (cm): men < 90, women < 85			
Q1	271	1.00		974	1.00		
Q2	539	1.13 (0.63–2.04)	0.679	666	1.00 (0.64–1.58)	0.994	0.069
Q3	760	1.33 (0.76–2.32)	0.325	458	1.50 (0.88–2.58)	0.139	
Q4	922	1.70 (0.93–3.09)	0.084	298	1.24 (0.62–2.51)	0.546	
HOMA-IR (quartiles)	BMI (kg/m^2^): ≥30			BMI (kg/m^2^): <30			
Q1	18	1.00		1227	1.00		
Q2	49	0.92 (0.10–8.40)	0.944	1156	1.01 (0.71–1.43)	0.966	0.160
Q3	112	1.55 (0.22–11.02)	0.660	1106	1.26 (0.86–1.85)	0.239	
Q4	228	1.85 (0.26–13.24)	0.542	992	1.38 (0.88–1.76)	0.164	
HOMA-IR (quartiles)	BF%: men ≥ 25, women ≥ 35				BF%: men < 25, women < 35		
Q1	462	1.00		783	1.00		
Q2	635	1.28 (0.78–2.10)	0.332	570	0.85 (0.51–1.42)	0.539	0.072
Q3	826	1.78 (1.09–2.90)	0.021	392	0.79 (0.43–1.48)	0.468	
Q4	957	1.91 (1.10–3.31)	0.022	263	1.00 (0.47–2.14)	0.996	

Models were adjusted for age, sex, educational status, cigarette smoking, drinking, history of hypertension and diabetes mellitus, FBG, SBP, TC, TG, WC, BMI, and BF% apart from the independent variable (WC, BMI, or BF%) of each model

*BF%* body fat percentage; *BMI* body mass index; *CI* confidence interval; *FBG* fasting blood glucose; *HOMA-IR* homeostasis model assessment-insulin resistance; *OR* odds ratio; *SBP* systolic blood pressure; *TC* total cholesterol; *TG* triglyceride; *WC* waist circumference

### Interaction of HOMA-IR with BF%, WC, and BMI

The measures of the interaction of different obesity indicators with high HOMA-IR are presented in Table 4. RERI and AP for interaction between high HOMA-IR and high BF% were 0.32 (0.04–0.60) and 0.33 (0.06–0.60), respectively, which indicated that the interaction was responsible for 0.32 RERI and accounted for 33% of the hypertension risk. However, we did not observe any significant interactions between obesity, defined according to WC (RERI: 0.15 [-0.23–0.50]; AP: 0.11 [-0.19–0.33]) or BMI (RERI: 0.65 [-0.04–1.46]; AP: 0.34 [-0.07–0.55]) and high HOMA-IR regarding hypertension.

**Table 4:** Interactions of HOMA-IR with BF%, WC, and BMI

	BF%: men ≥ 25, women ≥ 35			WC (cm): men ≥ 90, women ≥ 85			BMI (kg/m^2^): ≥30	
High HOMA-IR	RERI	AP		RERI	AP		RERI	AP
	0.32 (0.04–0.60)	0.33 (0.06–0.60)		0.15 (-0.20–0.51)	0.11 (-0.14–0.36)		0.65 (-0.04–1.46)	0.34 (-0.07–0.55)

Models were adjusted for age, sex, educational status, cigarette smoking, drinking, history of diabetes mellitus, FBG, TC, TG, BF%, WC, and BMI apart from the independent variable (WC, BMI, or BF%) of each model

*AP* attributable proportion due to interaction; *BF%* body fat percentage; *BMI* body mass index; *FBG* fasting blood glucose; *HOMA-IR* homeostasis model assessment-insulin resistance; *RERI* relative excess risk due to interaction; *TC* total cholesterol; *TG* triglyceride; *WC* waist circumference

Considering the sexual dimorphism in BF% distribution, we also performed subgroup analyses in men and women and divided the parameter BF% into three parts: truncal, leg, and arm BF%. High truncal, leg, and arm BF% values were defined as those above the respective 90th percentiles. Table 5 shows that high truncal BF% and high HOMA-IR accounted for a 40% (95% CI: 7–73%) risk of hypertension in women. Moreover, we found that 0.54 (95% CI: 0.04–1.05) RERI was attributed to the interaction between high leg BF% and high HOMA-IR, accounting for 45% (95% CI: 15–75%) risk of hypertension in women. However in men, there were no significant interactions between total or regional BF% and high HOMA-IR on hypertension. 

**Table 5:** Interactions of HOMA-IR with different distributions of BF% on hypertension by sex

	Men			Women	
BF%	High HOMA-IR			High HOMA-IR	
	RERI	AP		RERI	AP
High truncal BF%	-0.41 (-1.90–1.09)	-0.87 (-4.51–2.76)		0.42 (-0.02–0.87)	0.40 (0.07–0.73)
High leg BF%	-0.72 (-4.77–3.33)	-0.87 (-6.91–5.17)		0.54 (0.04–1.05)	0.45 (0.15–0.75)
High arm BF%	0.23 (-0.34–0.80)	0.57 (-0.43–1.58)		0.30 (-0.17–0.77)	0.27 (-0.10–0.64)

High truncal BF% was categorized as above the 90th percentile. High leg BF% was categorized as above the 90th percentile of the mean of the left and right legs. High arm BF% was categorized as above the 90th percentile of the mean BF% of the left and right arms

The models were adjusted for age, educational status, cigarette smoking, drinking, history of diabetes mellitus, FBG, TC, TG, WC, and BMI.

*AP* attributable proportion due to interaction; *BF%* body fat percentage; *BMI* body mass index; *FBG* fasting blood glucose; *HOMA-IR* homeostasis model assessment-insulin resistance; *RERI* relative excess risk due to interaction; *TC* total cholesterol; *TG* triglyceride; *WC* waist circumference

## Discussion

The major findings of our cross-sectional study suggest that the relationship between IR and the risk of hypertension is mediated by obesity. Moreover, an additive interaction was observed between BF% and IR on hypertension, especially in women with high HOMA-IR and high truncal BF% or high leg BF%.

Obesity plays a role in the onset and progression of IR [6, 23]. The coexistence of IR and obesity results in a substantial increase in the risk of hypertension. Therefore, when analyzing the relationship between IR and hypertension, most researchers have added common obesity indicators such as WC or BMI as potential confounders to the models. Our findings are generally in accordance with those of several studies in which the association between IR and hypertension disappeared after adjustment for obesity. For instance, in a prospective study with 8.9 years of follow-up, IR was positively associated with hypertension incidence among 1725 Iranian men, whereas this association was not significant when WC and BMI were included in the models [9]. Another cohort study [8] identified visceral obesity as a mediator of IR on increased hypertension risk, and increased visceral obesity, measured using dual-energy X-ray absorptiometry (DXA), explained 69.1% of the risk of incident hypertension associated with IR. A few reviews [14, 24, 25] also mentioned the role of obesity in the relationship between hypertension and IR. In addition, some studies have evaluated the relationship between IR and hypertension by sex stratification, as well as sex differences in these relationships suggesting that differences in body fat distribution affect this relationship [9, 26]. Another study found that IR is positively associated with a greater risk of incident hypertension among participants in overweight/obese BMI or high WC groups [27]. Therefore, general and central adiposity may have confounding or mediating effects on the association between IR and incident hypertension. However, other studies observed no modifying effects of obesity on this relationship. A recent meta-analysis of prospective studies provided evidence that IR is independently associated with subsequent risk of hypertension in the general population, which included original studies that considered at least one of the BMI and WC variables [5]. A 20-year follow-up longitudinal study [28] reported that BMI does not significantly modify the positive association between IR and the incidence of hypertension among Americans. Another prospective study consisting of 2814 Iranians also found that IR was significantly associated with the development of hypertension after considering BMI changes [29]. However, the above two prospective studies did not provide enough information to evaluate whether obesity indicators of WC and BF% mediated the association between IR and hypertension. Kaze et al. [27] examined the positive relationship between IR and incident hypertension; however, the researchers did not adjust for WC or BMI as confounders.

It is worth noting that BMI and WC are the two most common adjustment variables to represent obesity. In addition to these parameters being strongly correlated with IR, they are also closely associated with the risk of hypertension as confirmed by several longitudinal observational studies in different populations [30–32]. Recent cohort studies have demonstrated that BF% measured by BIA is independently associated with cardiovascular events [33], including hypertension [12]. Moreover, BF% is more strongly correlated with cardiovascular risk [33, 34] and hypertension [35] than with BMI or WC. Thus, whether BF% affects the relationship between IR and hypertension independent of BMI and WC needs to be confirmed. Our study found additive interactions between high HOMA-IR and obesity, defined as a high BF%, on hypertension. Interestingly, our study findings suggest that high truncal and leg BF% with high HOMA-IR has significant interactions with hypertension only in women, but not in men. The differences in the distribution of BF% between men and women might provide an explanation for this discrepancy; women had higher truncal BF% than men (36.65 vs. 28.21, *P* < 0.001), and leg BF% in women was higher than that in men (33.31 vs. 27.43, *P* < 0.001). A growing body of evidence supports that truncal adipose tissue has a significant impact on the development of IR and diabetes mellitus [36], and a study found that truncal fat-to-leg fat ratio is significantly associated with cardiovascular disease in patients with type 2 diabetes independently of BMI and WC [37]. Tillin et al. [38] also described that IR and truncal obesity account for the two-fold excess diabetes risk in Indian Asian and African Caribbean women, but not in men, in a cohort followed up for 20 years. The underlying explanation might be that truncal obesity causes an inflammatory state and leads to metabolic diseases such as hypertension and IR. However, the interactions of BF% with IR on hypertension risk have not been explored before as performed in the current study, perhaps because BF% measurements are time-consuming and expensive if using the gold standard approach of DXA. In addition, most studies tend to evaluate the relationship between total BF% and IR or metabolic disease, not considering it as a confounder to adjust or explore the interaction effects between IR and BF%. Our study may advance reasonable proposals to focus on both truncal BF% and IR in the development of hypertension. In addition to BMI, WC, and BF%, a body shape index (ABSI) and waist-to-height ratio (WHtR) serve as valuable indicators of obesity. ABSI was proposed based on a person’s WC and adjusting for their height and weight [39]. Higher ABSI have been associated with increased risks of mortality [39, 40], and hypertension [41]. Our findings indicate that elevated ABSI is a risk factor for hypertension, with a stronger association observed in males compared to females (Supplementary Table 1). However, in our study, there was no significant association between WHtR and hypertension after adjusting for traditional confounders (Supplementary Table 2). Future research should consider analyzing WHtR trends as a marker of changing central adiposity.

Our study may provide additional insights into the association between different obesity indicators and IR on hypertension risk. To our knowledge, this is the first study to consider that BF% has a confounding effect on the relationship between IR and the risk of hypertension, and we found for the first time that BF% mediates this relationship, especially in women with high truncal and leg BF% and high IR. However, the present study has certain limitations. First, as it was cross-sectional, we cannot assert causality, and the results require a longitudinal cohort study for verification. Second, BF% was measured using BIA instead of the gold standard approach of DXA. This might have introduced some bias. Nevertheless, existing research has shown a strong correlation between BIA-derived BF% and DXA in the general adult population [42]. Similarly, BIA-estimated WC shows a high correlation with direct manual measurement [43].Overall, BIA estimation of body composition is more suitable for large epidemiological studies where it can facilitate rapid screening of body composition metrics. However, BIA measures are dependent on several factors, including age, gender, ethnicity, overweight/obesity conditions, and the environment. Therefore, BIA measurements should be based on specific BIA equations tailored for different populations in the studies [44]. Our BIA devices, purchased from South Korea, are suitable for use in Asia and have specific calibration equations for the Chinese population. Third, as this study was performed in a Chinese rural population, the participants were relatively old (approximately 45% of participants were 60 years or older), and 23% had abnormal glucose tolerance; therefore, the generalizability of the study results to other ethnicities might be questionable.

## Conclusions

Our findings indicate that BF% modifies the association between IR and increased risk of hypertension in women with high truncal and leg BF%, but not in men.

### Electronic supplementary material

Below is the link to the electronic supplementary material.

Supplementary Material 1

## Data Availability

The datasets used and/or analysed during the study are available from the corresponding author on reasonable request.

## List of abbreviations

AP: attributable proportion
BF%: body fat percentage
BIA: bioelectrical impedance analysis
BMI: body mass index
CI: confidence interval
CNC-NX: China Northwest Natural Population Cohort, Ningxia Project
DBP: diastolic blood pressure
DXA: dual-energy X-ray absorptiometry
FBG: fasting blood glucose
HOMA-IR: homeostasis model assessment index
IR: insulin resistance
OR: odds ratio
RERI: relative excess risk
SBP: systolic blood pressure
WC: waist circumference
