# Supplementary material for: Additive interactions between obesity and insulin resistance on hypertension in a Chinese rural population
Source: BMC Public Health. 2023 Dec 15;23:2519. doi: 10.1186/s12889-023-17454-1 (PMC10724980; doi:10.1186/s12889-023-17454-1)
Supplement: Supplementary file 1 — Supplementary Material 1 [file 12889_2023_17454_MOESM1_ESM.docx]

**Supplementary table 1** Multivariable-adjusted logistic regression analysis of ABSI in relation to hypertension

|  | Total population | | Men | | Women | |
| --- | --- | --- | --- | --- | --- | --- |
|  | OR* (95% CI) | P-value | OR* (95% CI) | P-value | OR* (95% CI) | P-value |
| ABSI (quartiles) |  |  |  |  |  |  |
| Q1 | 1.00 (ref) |  | 1.00 (ref) |  | 1.00 (ref) |  |
| Q2 | 1.28 (1.04-1.58) | 0.021 | 1.68 (1.24-2.26) | 0.001 | 1.04 (0.83-1.31) | 0.739 |
| Q3 | 1.91 (1.34-2.72) | P<0.001 | 1.39 (1.07-1.81) | 0.013 | 1.37 (1.01-1.84) | 0.041 |
| Q4 | 2.69 (1.88-3.83) | P<0.001 | 1.78 (1.29-2.47) | 0.001 | 1.28 (0.97-1.69) | 0.081 |
| P for trend | P<0.001 | | P<0.001 | | 0.049 | |

Models adjusted for age, sex (only in total population), educational status, cigarette smoking, drinking, history of hypertension, diabetes mellitus and FBG, TC, and TG.

**Supplementary table 2** Multivariable-adjusted logistic regression analysis of WHtR in relation to hypertension

|  | **Model 1**  **OR (95% CI)** | **Model 2**  **OR (95% CI)** | **Model 3**  **OR (95% CI)** |
| --- | --- | --- | --- |
| WHtR | 2.74 (1.88-2.26) | 2.87 (2.15-3.82) | 1.03 (0.69-1.54) |

Data are presented as ORs and 95% CIs.

Model 1: crude. Model 2: adjusted for age, sex, educational status, cigarette smoking, drinking. Model 3: adjusted for all the factors in Model 2 and FBG, SBP, TC, and TG.
